# Incidence and case fatality of acute myocardial infarction in Korea, 2011-2020

**DOI:** 10.4178/epih.e2024002

**Published:** 2023-12-26

**Authors:** Yeeun Seo, Jenny Moon, Hyeok-Hee Lee, Hyeon Chang Kim, Fumie Kaneko, Sojung Shin, Eunji Kim, Jang-Whan Bae, Byeong-Keuk Kim, Seung Jun Lee, Min Kim, Hokyou Lee

**Affiliations:** 1 Department of Public Health, Yonsei University Graduate School, Seoul, Korea; 2 Department of Preventive Medicine, Yonsei University College of Medicine, Seoul, Korea; 3 Department of Internal Medicine, Yonsei University College of Medicine, Seoul, Korea; 4 Institute for Innovation in Digital Healthcare, Yonsei University, Seoul, Korea; 5 Department of Internal Medicine, Chungbuk National University College of Medicine, Cheongju, Korea; 6 Division of Cardiology, Department of Internal Medicine, Chungbuk National University Hospital, Cheongju, Korea; 7 Division of Cardiology, Severance Cardiovascular Hospital, Yonsei University Health System, Seoul, Korea

**Keywords:** Incidence, Case fatality rate, Acute myocardial infarction

## Abstract

**OBJECTIVES:**

Cardiovascular diseases are a leading cause of mortality worldwide, and acute myocardial infarction (AMI) is particularly fatal condition. We evaluated the incidence and case fatality rates of AMI in Korea from 2011 to 2020.

**METHODS:**

We utilized data from the National Health Insurance Services to calculate crude, age-standardized, and age-specific incidence rates, along with 30-day and 1-year case fatality rates, of AMI from 2011 to 2020. Age-standardized incidence rates were determined using direct standardization to the 2005 population.

**RESULTS:**

The crude incidence rate of AMI per 100,000 person-years consistently increased from 44.7 in 2011 to 68.3 in 2019, before decreasing slightly to 66.2 in 2020. The age-standardized incidence rate of AMI displayed a 19% rise from 2011 to 2019, followed by a slight decline in 2020. The increasing trend for AMI incidence was more pronounced in males than in females. Both 30-day and 1-year case fatality rates remained stable among younger individuals but showed a decrease among older individuals. There was a minor surge in case fatality in 2020, particularly among recurrent AMI cases.

**CONCLUSIONS:**

Over the past decade, the AMI incidence rate in Korea has consistently increased, with a slight downturn in 2020. The case fatality rate has remained relatively stable except for a minor increase in 2020. This study provides data for continuous surveillance, the implementation of targeted interventions, and the advancement of research aimed at AMI in Korea.

## GRAPHICAL ABSTRACT

[Fig f5-epih-46-e2024002]

## Key Message

This study observed the incidence rate of acute myocardial infarction using data from Korean National Health Insurance Service. From 2011 to 2019, the incidence rate increased, but there was a slight decrease in 2020. Additionally, the fatality rate remained relatively stable throughout the study period, except for an increase in 2020. The study emphasizes the importance of continuous monitoring and preventive strategies for effective management of acute myocardial infarction.

## INTRODUCTION

Cardiovascular diseases (CVDs) are a leading cause of mortality worldwide, with myocardial infarction representing a particularly significant global health issue [1]. While Western countries have traditionally reported high rates of CVD incidence and mortality rates, considerable reductions have declined considerably over recent decades [2-4]. In contrast, East Asian countries have exhibited relatively low incidence rates of ischemic heart diseases, including acute myocardial infarction (AMI) [5]. However, in Korea, an upward trend in the incidence rate of AMI has been reported recently. The increasing incidence and severity of AMI, especially among the growing elderly population, have raised concerns in recent decades [6,7].

In Korea, resources such as the Korean Statistical Information Service, the Korea National Health and Nutrition Examination Survey, and National Health Insurance Service (NHIS) are used to calculate the mortality and prevalence rates of CVD [8]. However, establishing a consistent monitoring system for CVD incidence in Korea poses a challenge due to the lack of a systematic disease surveillance system and annual data updates. Additionally, varying methodologies used in prior studies and the use of different datasets compound the difficulty in accurately assessing AMI incidence trends [6-9].

By developing a reliable methodology using the NHIS database, we aimed to estimate the incidence and case fatality rates of AMI in Korea and provide a comprehensive perspective on AMI trends over the past decade.

## MATERIALS AND METHODS

### Data source

We used a nationwide anonymized health information database of the NHIS, which is the single provider of mandatory health insurance for the entire Korean population. The National Health Insurance Big Data system includes socio-demographic information; hospital claims with International Classification of Diseases, 10th revision (ICD-10) coding; and fatality data for the entire Korean population [10]. Notably, the fatality data included in this study are not directly linked to the records maintained by Korea’s National Statistical Office.

### Ascertainment of acute myocardial infarction events

The data extraction was focused on patients admitted for AMI based on ICD-10 diagnosis codes (I21, I22, or I23), between January 1, 2002 and December 31, 2020. We retrieved all relevant health insurance claim records for these individuals. Health insurance claims data frequently associate multiple claims to a single disease event, necessitating the consolidation of various claim codes pertaining to drug prescriptions, diagnostic tests, and procedures. These codes can be scattered across multiple claims, complicating a comprehensive and accurate understanding of the disease episode. Consequently, we introduced the concept of a “hospitalization episode”, which is defined by two consecutive insurance claims, A and B. These claims are considered separate hospitalization episodes under two conditions: (1) if the gap between the initial dates of claims A and B exceeds 28 days, and (2) if the interval between the final date of claim A and the initial date of claim B is 3 days or more. This method enables a more effective capture of the complete range of patient treatment and outcomes associated with each distinct hospitalization episode.

Each hospitalization episode served as the subject for AMI event identification. We developed specific identification algorithms for the first and recurrent AMI events, as detailed in Table 1. Although both algorithms primarily relied on the use of ICD-10 diagnosis codes (I21, I22, and I23) for identification, the algorithm for recurrent events included additional rigorous criteria. Moreover, these diagnosis codes are supplemented with relevant diagnostic tests/or procedure codes.

To ensure the accuracy of these AMI identification algorithms, we conducted a retrospective review of medical records. This review included a total of 1,399 events from 24 hospitals throughout Korea, including 5 tertiary, 11 secondary, and 8 primary institutions. We established epidemiological adjudication criteria grounded on the Fourth Universal Definition of Myocardial Infarction (UDMI) albeit with modifications, to confirm the validity of each event identified by the algorithms [11]. The positive predictive values (PPVs) were calculated by dividing the number of algorithm-identified events adjudicated as true AMI cases by the total number of algorithm-identified events examined. PPVs were calculated separately for first and recurrent events, as well as by institution type (tertiary, secondary, and primary). The methodology for the design of these identification algorithms has been separately reported.

### Statistical analysis

We calculated both crude and age-standardized incidence rates of AMI per 100,000 person-years over the study period. Direct age-standardized rates were calculated using the 2005 Korean population to facilitate the comparison of yearly rates. To compare incidence rates between males and females, we calculated sex-specific incidence rates using similar methods, utilizing separate standard populations for each sex. Additionally, we calculated the case fatality rate of AMI in two forms: the 30-day fatality rate and the 1-year fatality rate. Across the period from 2011 to 2020, we computed both aggregate and age-stratified case fatality rates. The age groups were categorized into 40-64 years, 65-79 years, and 80 years or older. All statistical analyses were conducted using R version 4.0.3 (R Foundation for Statistical Computing, Vienna, Austria).

### Ethics statement

This study complied with the Declaration of Helsinki, and the study protocol was approved by the Institutional Review Board (IRB) of Yonsei University Health System, Seoul, Korea (#4-2022-0586). Informed consent was waived by the IRB. Informed consent was waived since this is a retrospective study of de-identified administrative data.

## RESULTS

### Number of acute myocardial infarction events, 2011-2020

Table 2 presents the number of AMI events identified between 2011 and 2020. During this period, 290,809 patients were admitted to the hospital due to AMI. The quantity of AMI events steadily rose from 22,398 in 2011 to 35,066 in 2019, marking an aggregate increase of 57.0%. However, in 2020, a slight downturn was observed with 33,988 events. The number of first AMI events mirrored the overall trend as they constitute the majority of total events. Notably, the share of recurrent AMI among all AMI events rose from 6.6% in 2011 to 8.7% in 2020.

When analyzed by sex, the total number of AMI events steadily increased for both males and females from 2011 to 2019, but slightly receded in 2020. The first and recurrent AMI events followed a similar pattern. Overall, the AMI events escalated more rapidly in males compared to females, resulting in an increase in the male-to-female ratio from 2.1 in 2011 to 2.7 by 2020 (Table 2).

### Age-stratified number of acute myocardial infarction events, 2011-2020

Table 3 displays the incidence of AMI events, stratified by age, from 2011 to 2020. In individuals under 20 years of age, the total number of incident AMI events was minimal fluctuating between 3 and 14 across the years. For those 20-29 years, the total number of AMI events varied with a high of 80 in 2020 and a low of 43 in 2012. In the 30-39 age group, the number peaked at 612 in 2016 and decreased slightly to 572 in 2020. The 40-49 age group, there was an increase from 2,334 events in 2011 to 2,873 in 2020. Similarly, the number of AMI events in those 50-59 age group saw number rise from 4,902 in 2011 to 6,990 in 2020. The 60-69 age group saw an increase from 5,014 events in 2011 to 9,047 in 2020, and individuals aged 70-79 saw their counts grow from 6,137 in 2011 to 7,850 in 2020. Those 80 years old and above was a significant surge in AMI events from 3,477 in 2011 to 6,567 in 2020. Across total, first, recurrent AMI events, increasing trends were particularly evident in the age group of 40-49 years, 50-59 years, 60-69 years, 70-79 years, and those aged 80 years old or above. Additionally, the number of AMI events for individuals under 20 years and those in the 20-29 age group remained considerably low.

According to Supplementary Materials 1 and 2, there were similar patterns were observed in AMI incidence across various age groups for both males and females over the year. In individuals younger than 20 age group, the number of AMI events remained consistently low. In contrast, older age group, especially those aged 60-69 years, 70-79 years, and over 80, showed an increased trend in incidence. This increasing trends was apparent not only in total number of AMI events but also in both first and recurrent AMI events, with the older age groups consistently exhibiting rising trends over the decade.

### Incidence rate of acute myocardial infarction, 2011-2020

**Figure 1.**
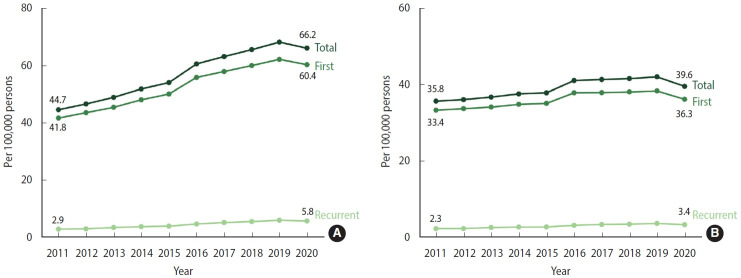
Incidence rate of acute myocardial infarction, 2011-2020. (A) Crude. (B) Age-standardized.

The crude incidence rate of AMI per 100,000 person-years demonstrated a consistent increase from 44.7 in 2011 to 68.3 in 2019, representing a cumulative increase of 54%. However, in 2020, this incidence rate declined slightly to 66.2 (Figure 1A, Supplementary Materials 3). The age-standardized incidence rate of AMI from 2011 to 2019 showed a steady increase, though this rise occurred more slowly compared to the crude incidence rate. In 2020, a slight decrease in this rate was observed (Figure 1B, Supplementary Materials 4). Given that most AMI events were first incidents, their incidence rate mirrored the total incidence rate. The rates of recurrent AMI incidence followed a similar pattern, though the extent of change was considerably smaller.

**Figure 2.**
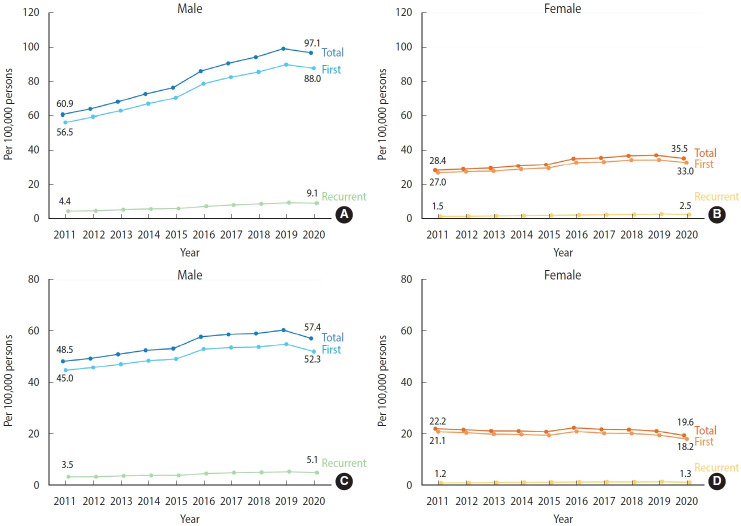
Sex-stratified incidence rate of acute myocardial infarction, 2011-2020. (A, B) Crude. (C, D) Age-standardized.

Sex-specific incidence rates of AMI presented comparable trends, although a significant sex-difference was evident in absolute levels. The crude incidence rate consistently increased from 2011 to 2019 for both sexes, with a slight decline observed in 2020 (Figure 2A and B, Supplementary Materials 5). However, the age-standardized incidence rates of AMI showed different trends between sexes. In males, the age-standardized incidence rate rose steadily from 2011 to 2019, with a minor tapering off in 2020 (Figure 2C, Supplementary Materials 6). In contrast, the age-standardized incidence rate in females displayed only a minimal change compared to males (Figure 2D, Supplementary Materials 6).

### Case fatality rate of acute myocardial infarction, 2011-2020

**Figure 3.**
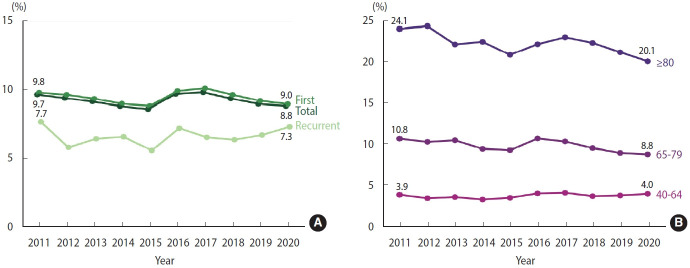
30-Day case fatality rate of acute myocardial infarction, 2011-2020. (A) Crude. (B) Age-stratified.

The overall 30-day fatality rate for total and first AMI events remained steady throughout the study period, at around 8-9%. The 30-day fatality rate for recurrent AMI remained at 5-7% from 2011 to 2019 (Figure 3A, Supplementary Materials 7). When stratified by age, the 30-day fatality rate gradually decreased in those aged 80 over and 65-79 from 2011 to 2020. However, for those aged 40-64 years, the rate remained relatively stable (Figure 3B, Supplementary Materials 8).

**Figure 4.**
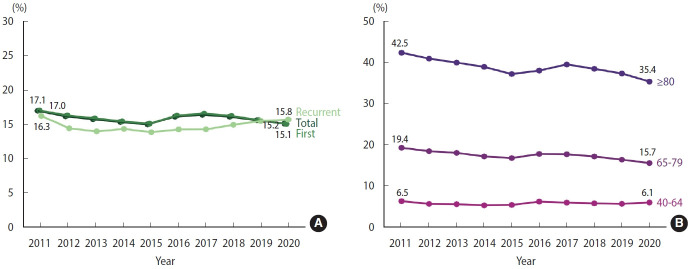
1-Year case fatality rate of acute myocardial infarction, 2011-2020. (A) Crude. (B) Age-stratified.

The overall 1-year fatality rate for both total and first AMI remained stable at around 15-17% from 2011 to 2020. The recurrent AMI rate steadily remained around 13-16% from 2012 to 2020 (Figure 4A, Supplementary Materials 9). Similar to the 30-day case fatality rate, the 1-year fatality rate of AMI was higher in older age groups compared to younger age group. The 1-year fatality rate of AMI for those aged 80 years and older and 65-79 years steadily decreased from 2011 to 2020. In contrast, the fatality rate of AMI for those aged 40-64 years remained relatively stable from 2011 to 2020 (Figure 4B, Supplementary Materials 10).

### Positive predictive values of the identification algorithms for acute myocardial infarction events

The PPVs of the algorithms used to identify first and recurrent AMI events are presented for in different types of hospitals in Supplementary Materials 11. The PPV for the first AMI identification algorithm was highest in tertiary hospitals at 94.7%, followed by secondary hospitals at 92.1%, and was lowest in primary hospitals at 42.1%. When fatalities where the cause of death was recorded as AMI were considered as positive events, an increase in PPVs was observed across all hospital types: 95.0% in tertiary hospitals, 92.9% in secondary hospitals, and 51.2% in primary hospitals. The crude pooled PPV reached 88.1%. When weighted according to the medical institution first visited and the highest-level medical institution during the episode, the PPV approximated 92.0% and 92.2%, respectively. When AMI-related fatalities were considered as positive events, the corresponding PPVs were 92.8% and 93.0%, respectively.

The recurrent AMI identification algorithm exhibited a PPV of 81.5% in tertiary institutions and 75.0% in secondary institutions. The rate could not be properly evaluated in primary institutions, since there was only one identified AMI case. When AMI-related fatalities were considered as positive events, PPVs did not change across all institution types. The crude pooled PPV of the recurrent AMI identification algorithm was 77.1%, and the weighted according to the medical institution first visited and the highest-level medical institution during the episode, the PPV approximated 77.8% and 78.2%, respectively.

## DISCUSSION

This study provides a comprehensive analysis of the decade-long trends in the incidence and case fatality rates of AMI in Korea. From 2011 to 2019, a significant 57.0% increase was observed in the total number of AMI events, followed by a slight reduction in 2020. While recurrent events comprised a small portion of the total AMI incidents, their proportion has steadily increased, particularly pronounced among males patients, resulting in a widening male-to-female ratio. During the same period, both crude and the age-standardized AMI incidence rates increase by 53% and 18%, respectively, although a minor decrease was recorded in 2020. The AMI incidence rate increased for both sexes; however, the rise was more pronounced among males. Despite a decline in AMI case fatality rates among older patients, a notable upturn was observed across all age groups in 2020.

Other countries have also conducted studies on the incidence rate of AMI. In Japan, various data sources such as local or regional registries, community cohort studies, and medical institution network research were utilized to measure the incidence rates of myocardial infarction. These studies, the diagnostic criteria primarily relied on the WHO-MONICA standards. However, in the most recent medical institution-based research, the UDMI was adopted to confirm the incidence of MI [5,12-14]. The MIYAGIAMI Registry study in Japan revealed that the incidence rate of AMI increased from 7.4 in 1979 to 27.0 in 2008 over a period of 30 years. The overall in-hospital mortality (age-adjusted) decreased from 20.0% in 1979 to 7.8% in 2008 [12]. In the Tokyo metropolitan area of Japan, the incidence rate of AMI remained consistent over an 11-year period from 2006 to 2016, unchanged from 41.3 to 40.7 [5]. Using the Shiga Stroke and Heart Attack Registry, this study to examine the incidence and in-hospital mortality of AMI, as defined by the UDMI, from January 2014 to December 2015. The age-adjusted incidence rate was 61.9 per 100,000 person-years, and the incidence of AMI increased with age for both sexes, peaking in individuals aged 85 years and older. In-hospital mortality among ST-elevation myocardial infarction patients remained elevated [14]. In Taiwan, using the Taiwan National Health Insurance Research Database (1999-2008), patients aged 18 years and older hospitalized for AMI were identified. The age-adjusted incidence rate of AMI increased from 28.0 per 100,000 person-years in 1999 to 44.4 in 2008. The in-hospital mortality rate decreased from 15.9% in 1999 to 12.3% in 2008 [15]. Using the Taiwan National Health Insurance Research Database, a study identified a total of 144,634 patients hospitalized for AMI from 1997 to 2011. The incidence rate of AMI increased from 30 per 100,000 persons in 1997 to 42 in 2011 [16]. Lastly, using the Taiwan National Health Insurance Research Database, a study examining 100,570 adult patients hospitalized for AMI from 2009 to 2015 and examined the temporal trends in the incidence of AMI. Overall, the age-adjusted and sex-adjusted incidence of AMI remained relatively stable, changing from 49.8 in 2009 to 50.7 in 2015. However, an increase was observed in the incidence of AMI among adults under 55 years old, with a 30.3% rise in young males and a 29.4% rise in young females, while the incidence in other age groups either decreased or remained unchanged [17]. Both countries have similar disease distribution patterns and healthcare systems to Korea. They also seek to identify the patterns in incidence rates, primarily focusing on age-standardized incidence rates. However, due to variations in the standard populations selected for each study, international comparison of age-standardized incidence rates was not possible.

The strength of this study is that it is a nationwide study that provides a comprehensive examination of the trends in AMI incidence and case fatality rates over a 10-year period in Korea. Additionally, the study also utilized a large database, the Korean National Health Insurance Service, which covers approximately 97% of the Korean population, thereby ensuring a representative sample. Furthermore, the study analyzed the trends by sex and age groups, providing a detailed understanding of the differences in AMI incidence and case fatality rates across population groups.

Our study had several limitations that warrant consideration. Firstly, the use of health insurance claim data for estimating incidence rates may not capture patients who did not utilize healthcare services or incidence cases outside the country. Additionally, the evolution of diagnostic tools and modifications in health insurance and healthcare systems can influence the estimated incidence rates. Therefore, it is important to conduct complementary community-based studies should be complemented to ascertain the magnitude of such cases that go unreported in the health insurance system. Secondly, despite our efforts to maximize validity by incorporating information on interventions and treatments beyond diagnostic codes, we were unable to access test results. To address this, retrospective medical records to determine the PPV and identify influential factors. Consequently, we established a PPV of 92.0%, a figure comparable to findings from similar international studies, signifying its relevance in monitoring and understanding AMI trends. Thirdly, the precise identification of AMI subtypes and the accurate diagnosis of AMI are significant challenges. Addressing these requires the collection of detailed clinical information and test results, underscoring the necessity for hospital-based registry studies. This highlights the need for hospital-based registry studies, which enable comprehensive data collection and access to complete clinical information and test results.

## CONCLUSION

This nationwide study provides critical insights into the trends of AMI incidence and case fatality rates in Korea, leveraging a large-scale database and a detailed analysis of trends stratified by sex and age groups. While Korea is among the countries with relatively low AMI incidence and fatality rates, the increasing number of AMI cases and incidence rate necessitate strategies to reduce the disease burden. Moreover, with nearly 10% of AMI patients dying within a month and another 20% within a year of the event, efforts to reduce these numbers are equally important. This study, through its detailed investigation of nationwide AMI trends, serves multiple purposes. It provides clinicians and researchers by providing comprehensive data on AMI, which can be used for further research. It assists healthcare policymakers in formulating effective plans for tackling AMI. Moreover, it helps the public understand the importance of AMI prevention and management. Ultimately, the insights gained from this study aim to enhance cardiovascular disease treatment and reduce its burden on society.

## Figures and Tables

**Figure f5-epih-46-e2024002:**
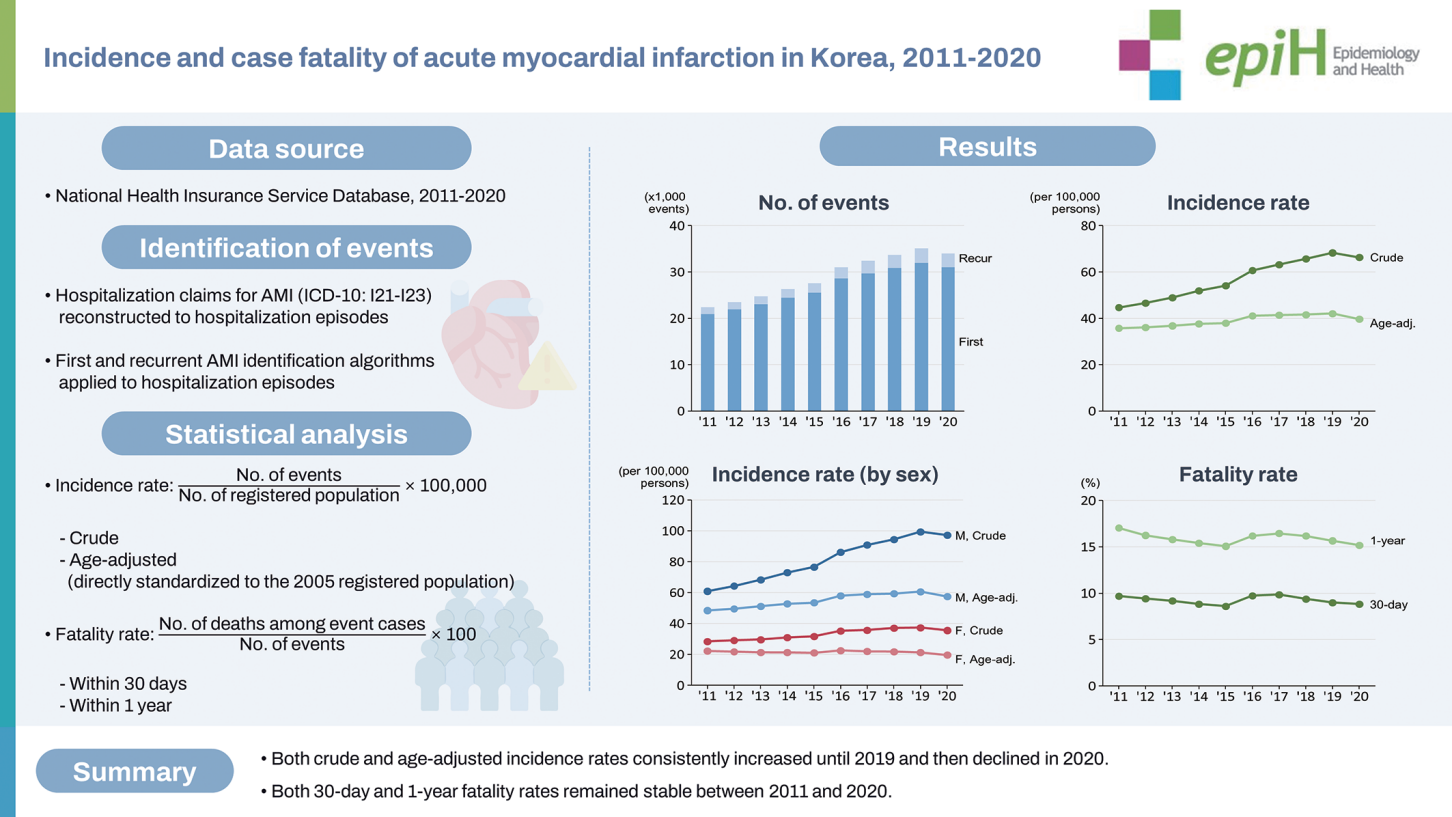


**Table 1.** Identification algorithms for first and recurrent AMI events

Event	Diagnosis codes (ICD-10)	Identification algorithm
First	Primary, I21-I23 (+)	ECG or cardiac enzyme test or CAG or PCI/CABG or death
	Primary, I21-I23 (-); Secondary or lower, I21-I23 (+)	CAG or PCI/CABG
Recurrent	All, I21-I23 (+)	PCI/CABG and (episode length ≥3 days or death)

AMI, acute myocardial infarction; ICD-10, International Classification of Diseases, 10th revision; ECG, electrocardiogram; CAG, coronary angiography; PCI, percutaneous coronary intervention; CABG, coronary artery bypass grafting.

**Table 2.** Number of acute myocardial infarction events, 2011-2020

Sex	2011	2012	2013	2014	2015	2016	2017	2018	2019	2020
Both sexes										
Total	22,398	23,505	24,784	26,367	27,585	31,017	32,405	33,694	35,066	33,988
First	20,930	21,974	23,020	24,445	25,563	28,597	29,723	30,829	31,962	31,017
Recurrent	1,468	1,531	1,764	1,922	2,022	2,420	2,682	2,865	3,104	2,971
Male										
Total	15,281	16,182	17,289	18,517	19,510	21,998	23,231	24,181	25,464	24,852
First	14,181	15,036	15,963	17,075	18,003	20,148	21,167	21,959	23,068	22,521
Recurrent	1,100	1,146	1,326	1,442	1,507	1,850	2,064	2,222	2,396	2,331
Female										
Total	7,117	7,323	7,495	7,850	8,075	9,019	9,174	9,513	9,602	9,136
First	6,749	6,938	7,057	7,370	7,560	8,449	8,556	8,870	8,894	8,496
Recurrent	368	385	438	480	515	570	618	643	708	640

**Table 3.** Age-stratified number of acute myocardial infarction events, 2011-2020

Age (yr)	2011	2012	2013	2014	2015	2016	2017	2018	2019	2020
Total										
<20	11	9	3	3	14	13	5	11	12	9
20-29	48	43	47	51	59	65	54	70	77	80
30-39	475	494	563	541	518	612	608	607	602	572
40-49	2,334	2,482	2,680	2,760	2,752	2,986	3,017	2,979	3,133	2,873
50-59	4,902	5,111	5,415	5,805	6,017	6,589	6,997	7,060	7,270	6,990
60-69	5,014	5,290	5,543	5,992	6,482	7,479	7,834	8,401	8,871	9,047
70-79	6,137	6,369	6,597	6,799	7,083	7,729	7,935	8,152	8,346	7,850
≥80	3,477	3,707	3,936	4,416	4,660	5,544	5,955	6,414	6,755	6,567
First										
<20	11	9	3	3	14	11	4	11	12	9
20-29	47	40	46	50	53	63	51	68	74	78
30-39	449	461	536	515	495	580	577	574	570	547
40-49	2,184	2,343	2,526	2,592	2,589	2,793	2,805	2,813	2,921	2,697
50-59	4,565	4,760	4,995	5,402	5,574	6,055	6,423	6,458	6,687	6,436
60-69	4,659	4,909	5,098	5,483	5,946	6,830	7,128	7,605	7,982	8,157
70-79	5,699	5,935	6,121	6,260	6,530	7,099	7,176	7,324	7,474	7,075
≥80	3,316	3,517	3,695	4,140	4,362	5,166	5,559	5,976	6,242	6,021
Recurrent										
<20	-	-	-	-	-	2	1	-	-	-
20-29	1	3	1	1	6	2	3	2	3	2
30-39	26	33	27	26	23	32	31	33	32	25
40-49	150	139	154	168	163	193	212	166	212	179
50-59	337	351	420	403	443	534	574	602	583	554
60-69	355	381	445	509	536	649	706	796	889	890
70-79	438	434	476	539	553	630	759	828	872	775
≥80	161	190	241	276	298	378	396	438	513	546

## Supplementary materials

Supplementary Material 1. Age-stratified incidence AMI event in males, 2011-2020

Supplementary Material 2. Age-stratified incidence AMI event in females, 2011-2020

Supplementary Material 3. Crude incidence rate of AMI per 100,000 person-years in 2011-2020

Supplementary Material 4. Age-adjusted incidence rate of AMI per 100,000 person-years in 2011-2020

Supplementary Material 5. Crude sex-specific incidence rate of AMI per 100,000 person-years in 2011-2020

Supplementary Material 6. Age-adjusted and sex-specific incidence rate of AMI per 100,000 person-years in 2011-2020

Supplementary Material 7. Thirty-day case fatality of AMI, 2011-2020 (%)

Supplementary Material 8. Age-stratified thirty-day case fatality of AMI, 2011-2020 (%)

Supplementary Material 9. One-year case fatality of AMI, 2011-2020 (%)

Supplementary Material 10. Age-stratified one-year case fatality of AMI, 2011-2020 (%)

Supplementary Material 11. Positive predictive value of the working definition for AMI event
