# Supplementary material for: Incidence and case fatality of acute myocardial infarction in Korea, 2011-2020
Source: Epidemiol Health. 2023 Dec 26;46:e2024002. doi: 10.4178/epih.e2024002 (PMC10928467; doi:10.4178/epih.e2024002)
Supplement: Supplementary Material 1. — Age-stratified incidence AMI event in males, 2011-2020 [file epih-46-e2024002-Supplementary-1.docx]

Supplementary Material 1. Age-stratified incidence AMI event in males, 2011-2020

| **Male** | | **Year** | | | | | | | | | |
| --- | --- | --- | --- | --- | --- | --- | --- | --- | --- | --- | --- |
|  |  | **2011** | **2012** | **2013** | **2014** | **2015** | **2016** | **2017** | **2018** | **2019** | **2020** |
| **Total** | | | | | | | | | | | |
|  | <20 | 8 | 8 | 3 | 2 | 10 | 9 | 5 | 9 | 10 | 9 |
|  | 20-29 | 44 | 40 | 43 | 49 | 51 | 60 | 49 | 62 | 66 | 71 |
|  | 30-39 | 446 | 466 | 535 | 506 | 487 | 565 | 562 | 563 | 561 | 526 |
|  | 40-49 | 2,136 | 2,291 | 2,500 | 2,557 | 2,534 | 2,731 | 2,793 | 2,701 | 2,833 | 2,587 |
|  | 50-59 | 4,289 | 4,488 | 4,819 | 5,151 | 5,370 | 5,871 | 6,220 | 6,207 | 6,461 | 6,273 |
|  | 60-69 | 3,715 | 4,006 | 4,199 | 4,650 | 5,096 | 5,873 | 6,190 | 6,710 | 7,168 | 7,302 |
|  | 70-79 | 3,378 | 3,511 | 3,700 | 3,942 | 4,185 | 4,623 | 4,880 | 5,163 | 5,375 | 5,093 |
|  | ≥80 | 1,265 | 1,372 | 1,490 | 1,660 | 1,777 | 2,266 | 2,532 | 2,766 | 2,990 | 2,991 |
| **First** | | | | | | | | | | | |
|  | <20 | 8 | 8 | 3 | 2 | 10 | 9 | 4 | 9 | 10 | 9 |
|  | 20-29 | 43 | 37 | 42 | 48 | 46 | 58 | 46 | 60 | 63 | 69 |
|  | 30-39 | 421 | 436 | 509 | 481 | 464 | 534 | 533 | 533 | 532 | 502 |
|  | 40-49 | 1,997 | 2,162 | 2,355 | 2,400 | 2,382 | 2,552 | 2,594 | 2,541 | 2,635 | 2,416 |
|  | 50-59 | 3,986 | 4,172 | 4,436 | 4,780 | 4,958 | 5,379 | 5,694 | 5,655 | 5,923 | 5,755 |
|  | 60-69 | 3,440 | 3,699 | 3,848 | 4,241 | 4,656 | 5,335 | 5,598 | 6,037 | 6,422 | 6,542 |
|  | 70-79 | 3,100 | 3,255 | 3,405 | 3,590 | 3,849 | 4,217 | 4,377 | 4,586 | 4,764 | 4,536 |
|  | ≥80 | 1,186 | 1,267 | 1,365 | 1,533 | 1,638 | 2,064 | 2,321 | 2,538 | 2,719 | 2,692 |
| **Recurrent** | | | | | | | | | | | |
|  | <20 | - | - | - | - | - | - | 1 | - | - | - |
|  | 20-29 | 1 | 3 | 1 | 1 | 5 | 2 | 3 | 2 | 3 | 2 |
|  | 30-39 | 25 | 30 | 26 | 25 | 23 | 31 | 29 | 30 | 29 | 24 |
|  | 40-49 | 139 | 129 | 145 | 157 | 152 | 179 | 199 | 160 | 198 | 171 |
|  | 50-59 | 303 | 316 | 383 | 371 | 412 | 492 | 526 | 552 | 538 | 518 |
|  | 60-69 | 275 | 307 | 351 | 409 | 440 | 538 | 592 | 673 | 746 | 760 |
|  | 70-79 | 278 | 256 | 295 | 352 | 336 | 406 | 503 | 577 | 611 | 557 |
|  | ≥80 | 79 | 105 | 125 | 127 | 139 | 202 | 211 | 228 | 271 | 299 |
