# Supplementary material for: Incidence and case fatality of acute myocardial infarction in Korea, 2011-2020
Source: Epidemiol Health. 2023 Dec 26;46:e2024002. doi: 10.4178/epih.e2024002 (PMC10928467; doi:10.4178/epih.e2024002)
Supplement: Supplementary Material 2. — Age-stratified incidence AMI event in females, 2011-2020 [file epih-46-e2024002-Supplementary-2.docx]

Supplementary Material 2. Age-stratified incidence AMI event in females, 2011-2020

| **Female** | | **Year** | | | | | | | | | |
| --- | --- | --- | --- | --- | --- | --- | --- | --- | --- | --- | --- |
|  |  | **2011** | **2012** | **2013** | **2014** | **2015** | **2016** | **2017** | **2018** | **2019** | **2020** |
| **Total** | | | | | | | | | | | |
|  | <20 | 3 | 1 | - | 1 | 4 | 4 | 0 | 2 | 2 | 0 |
|  | 20-29 | 4 | 3 | 4 | 2 | 8 | 5 | 5 | 8 | 11 | 9 |
|  | 30-39 | 29 | 28 | 28 | 35 | 31 | 47 | 46 | 44 | 41 | 46 |
|  | 40-49 | 198 | 191 | 180 | 203 | 218 | 255 | 224 | 278 | 300 | 286 |
|  | 50-59 | 613 | 623 | 596 | 654 | 647 | 718 | 777 | 853 | 809 | 717 |
|  | 60-69 | 1,299 | 1,284 | 1,344 | 1,342 | 1,386 | 1,606 | 1,644 | 1,691 | 1,703 | 1,745 |
|  | 70-79 | 2,759 | 2,858 | 2,897 | 2,857 | 2,898 | 3,106 | 3,055 | 2,989 | 2,971 | 2,757 |
|  | ≥80 | 2,212 | 2,335 | 2,446 | 2,756 | 2,883 | 3,278 | 3,423 | 3,648 | 3,765 | 3,576 |
| **First** | | | | | | | | | | | |
|  | <20 | 3 | 1 | - | 1 | 4 | 2 | - | 2 | 2 | - |
|  | 20-29 | 4 | 3 | 4 | 2 | 7 | 5 | 5 | 8 | 11 | 9 |
|  | 30-39 | 28 | 25 | 27 | 34 | 31 | 46 | 44 | 41 | 38 | 45 |
|  | 40-49 | 187 | 181 | 171 | 192 | 207 | 241 | 211 | 272 | 286 | 278 |
|  | 50-59 | 579 | 588 | 559 | 622 | 616 | 676 | 729 | 803 | 764 | 681 |
|  | 60-69 | 1,219 | 1,210 | 1,250 | 1,242 | 1,290 | 1,495 | 1,530 | 1,568 | 1,560 | 1,615 |
|  | 70-79 | 2,599 | 2,680 | 2,716 | 2,670 | 2,681 | 2,882 | 2,799 | 2,738 | 2,710 | 2,539 |
|  | ≥80 | 2,130 | 2,250 | 2,330 | 2,607 | 2,724 | 3,102 | 3,238 | 3,438 | 3,523 | 3,329 |
| **Recurrent** | | | | | | | | | | | |
|  | <20 | - | - | - | - | - | 2 | - | - | - | - |
|  | 20-29 | - | - | - | - | 1 | - | - | - | - | - |
|  | 30-39 | 1 | 3 | 1 | 1 | - | 1 | 2 | 3 | 3 | 1 |
|  | 40-49 | 11 | 10 | 9 | 11 | 11 | 14 | 13 | 6 | 14 | 8 |
|  | 50-59 | 34 | 35 | 37 | 32 | 31 | 42 | 48 | 50 | 45 | 36 |
|  | 60-69 | 80 | 74 | 94 | 100 | 96 | 111 | 114 | 123 | 143 | 130 |
|  | 70-79 | 160 | 178 | 181 | 187 | 217 | 224 | 256 | 251 | 261 | 218 |
|  | ≥80 | 82 | 85 | 116 | 149 | 159 | 176 | 185 | 210 | 242 | 247 |
