# Supplementary material for: Incidence and case fatality of acute myocardial infarction in Korea, 2011-2020
Source: Epidemiol Health. 2023 Dec 26;46:e2024002. doi: 10.4178/epih.e2024002 (PMC10928467; doi:10.4178/epih.e2024002)
Supplement: Supplementary Material 3. — Crude incidence rate of AMI per 100,000 person-years in 2011-2020 [file epih-46-e2024002-Supplementary-3.docx]

Supplementary Material 3. Crude incidence rate of AMI per 100,000 person-years in 2011-2020

| **Characteristics of AMI** | **Year** | | | | | | | | | |
| --- | --- | --- | --- | --- | --- | --- | --- | --- | --- | --- |
|  | **2011** | **2012** | **2013** | **2014** | **2015** | **2016** | **2017** | **2018** | **2019** | **2020** |
| Total | 44.7 | 46.7 | 49.0 | 51.9 | 54.1 | 60.7 | 63.3 | 65.7 | 68.3 | 66.2 |
| First | 41.8 | 43.6 | 45.5 | 48.2 | 50.2 | 55.9 | 58.0 | 60.1 | 62.3 | 60.4 |
| Recurrent | 2.9 | 3.0 | 3.5 | 3.8 | 4.0 | 4.7 | 5.2 | 5.6 | 6.0 | 5.8 |
