# Supplementary material for: Incidence and case fatality of acute myocardial infarction in Korea, 2011-2020
Source: Epidemiol Health. 2023 Dec 26;46:e2024002. doi: 10.4178/epih.e2024002 (PMC10928467; doi:10.4178/epih.e2024002)
Supplement: Supplementary Material 4. — Age-adjusted incidence rate of AMI per 100,000 person-years in 2011-2020 [file epih-46-e2024002-Supplementary-4.docx]

Supplementary Material 4. Age-adjusted incidence rate of AMI per 100,000 person-years in 2011-2020

| **Characteristics of AMI** | **Year** | | | | | | | | | |
| --- | --- | --- | --- | --- | --- | --- | --- | --- | --- | --- |
|  | **2011** | **2012** | **2013** | **2014** | **2015** | **2016** | **2017** | **2018** | **2019** | **2020** |
| Total | 35.8 | 36.2 | 36.8 | 37.7 | 37.9 | 41.2 | 41.4 | 41.7 | 42.1 | 39.6 |
| First | 33.4 | 33.8 | 34.2 | 34.9 | 35.2 | 38.0 | 38.0 | 38.2 | 38.4 | 36.3 |
| Recurrent | 2.3 | 2.4 | 2.6 | 2.7 | 2.8 | 3.2 | 3.4 | 3.5 | 3.7 | 3.4 |
