# Supplementary material for: Incidence and case fatality of acute myocardial infarction in Korea, 2011-2020
Source: Epidemiol Health. 2023 Dec 26;46:e2024002. doi: 10.4178/epih.e2024002 (PMC10928467; doi:10.4178/epih.e2024002)
Supplement: Supplementary Material 5. — Crude sex-specific incidence rate of AMI per 100,000 person-years in 2011-2020 [file epih-46-e2024002-Supplementary-5.docx]

Supplementary Material 5. Crude sex-specific incidence rate of AMI per 100,000 person-years in 2011-2020

| **Sex** | **Year** | | | | | | | | | |
| --- | --- | --- | --- | --- | --- | --- | --- | --- | --- | --- |
|  | **2011** | **2012** | **2013** | **2014** | **2015** | **2016** | **2017** | **2018** | **2019** | **2020** |
| **Male** |  |  |  |  |  |  |  |  |  |  |
| Total | 60.9 | 64.2 | 68.4 | 73.0 | 76.6 | 86.2 | 90.8 | 94.4 | 99.4 | 97.1 |
| First | 56.5 | 59.7 | 63.1 | 67.3 | 70.7 | 78.9 | 82.8 | 85.8 | 90.1 | 88.0 |
| Recurrent | 4.4 | 4.5 | 5.2 | 5.7 | 5.9 | 7.2 | 8.1 | 8.7 | 9.4 | 9.1 |
| **Female** |  |  |  |  |  |  |  |  |  |  |
| Total | 28.4 | 29.1 | 29.7 | 30.9 | 31.7 | 35.3 | 35.8 | 37.0 | 37.3 | 35.5 |
| First | 27.0 | 27.6 | 27.9 | 29.0 | 29.7 | 33.0 | 33.4 | 34.5 | 34.6 | 33.0 |
| Recurrent | 1.5 | 1.5 | 1.7 | 1.9 | 2.0 | 2.2 | 2.4 | 2.5 | 2.8 | 2.5 |
