# Supplementary material for: Incidence and case fatality of acute myocardial infarction in Korea, 2011-2020
Source: Epidemiol Health. 2023 Dec 26;46:e2024002. doi: 10.4178/epih.e2024002 (PMC10928467; doi:10.4178/epih.e2024002)
Supplement: Supplementary Material 6. — Age-adjusted and sex-specific incidence rate of AMI per 100,000 person-years in 2011-2020 [file epih-46-e2024002-Supplementary-6.docx]

Supplementary Material 6. Age-adjusted and sex-specific incidence rate of AMI per 100,000 person-years in 2011-2020

| **Sex** | **Year** | | | | | | | | | |
| --- | --- | --- | --- | --- | --- | --- | --- | --- | --- | --- |
|  | **2011** | **2012** | **2013** | **2014** | **2015** | **2016** | **2017** | **2018** | **2019** | **2020** |
| **Male** |  |  |  |  |  |  |  |  |  |  |
| Total | 48.5 | 49.6 | 51.2 | 52.8 | 53.4 | 58.0 | 59.0 | 59.3 | 60.7 | 57.4 |
| First | 45.0 | 46.1 | 47.4 | 48.7 | 49.4 | 53.3 | 53.9 | 54.1 | 55.2 | 52.3 |
| Recurrent | 3.5 | 3.5 | 3.9 | 4.0 | 4.0 | 4.8 | 5.1 | 5.2 | 5.5 | 5.1 |
| **Female** |  |  |  |  |  |  |  |  |  |  |
| Total | 22.2 | 21.8 | 21.4 | 21.3 | 21.0 | 22.6 | 22.0 | 21.9 | 21.3 | 19.6 |
| First | 21.1 | 20.7 | 20.1 | 20.0 | 19.7 | 21.2 | 20.5 | 20.4 | 19.7 | 18.2 |
| Recurrent | 1.2 | 1.2 | 1.3 | 1.3 | 1.4 | 1.4 | 1.5 | 1.5 | 1.6 | 1.3 |
