# Supplementary material for: Incidence and case fatality of acute myocardial infarction in Korea, 2011-2020
Source: Epidemiol Health. 2023 Dec 26;46:e2024002. doi: 10.4178/epih.e2024002 (PMC10928467; doi:10.4178/epih.e2024002)
Supplement: Supplementary Material 7. — Thirty-day case fatality of AMI, 2011-2020 (%) [file epih-46-e2024002-Supplementary-7.docx]

Supplementary Material 7. Thirty-day case fatality of AMI, 2011-2020 (%)

| **Characteristics of AMI** | **Year** | | | | | | | | | |
| --- | --- | --- | --- | --- | --- | --- | --- | --- | --- | --- |
|  | **2011** | **2012** | **2013** | **2014** | **2015** | **2016** | **2017** | **2018** | **2019** | **2020** |
| Total | 9.7 | 9.4 | 9.2 | 8.8 | 8.6 | 9.7 | 9.9 | 9.4 | 9.0 | 8.8 |
| First | 9.8 | 9.7 | 9.4 | 9.0 | 8.8 | 9.9 | 10.2 | 9.7 | 9.2 | 9.0 |
| Recurrent | 7.7 | 5.8 | 6.5 | 6.6 | 5.6 | 7.2 | 6.6 | 6.4 | 6.7 | 7.3 |
