# Supplementary material for: Incidence and case fatality of acute myocardial infarction in Korea, 2011-2020
Source: Epidemiol Health. 2023 Dec 26;46:e2024002. doi: 10.4178/epih.e2024002 (PMC10928467; doi:10.4178/epih.e2024002)
Supplement: Supplementary Material 8. — Age-stratified thirty-day case fatality of AMI, 2011-2020 (%) [file epih-46-e2024002-Supplementary-8.docx]

Supplementary Material 8. Age-stratified thirty-day case fatality of AMI, 2011-2020 (%)

| **Age, years** | **Year** | | | | | | | | | |
| --- | --- | --- | --- | --- | --- | --- | --- | --- | --- | --- |
|  | **2011** | **2012** | **2013** | **2014** | **2015** | **2016** | **2017** | **2018** | **2019** | **2020** |
| > 80 | 24.1 | 24.4 | 22.2 | 22.6 | 20.9 | 22.3 | 23.1 | 22.4 | 21.3 | 20.1 |
| 65-79 | 10.8 | 10.4 | 10.6 | 9.5 | 9.3 | 10.8 | 10.4 | 9.6 | 9.0 | 8.8 |
| 40-64 | 3.9 | 3.5 | 3.6 | 3.3 | 3.5 | 4.1 | 4.2 | 3.7 | 3.8 | 4.0 |
