# Supplementary material for: Incidence and case fatality of acute myocardial infarction in Korea, 2011-2020
Source: Epidemiol Health. 2023 Dec 26;46:e2024002. doi: 10.4178/epih.e2024002 (PMC10928467; doi:10.4178/epih.e2024002)
Supplement: Supplementary Material 9. — One-year case fatality of AMI, 2011-2020 (%) [file epih-46-e2024002-Supplementary-9.docx]

Supplementary Material 9. One-year case fatality of AMI, 2011-2020 (%)

| **Characteristics of AMI** | **Year** | | | | | | | | | |
| --- | --- | --- | --- | --- | --- | --- | --- | --- | --- | --- |
|  | **2011** | **2012** | **2013** | **2014** | **2015** | **2016** | **2017** | **2018** | **2019** | **2020** |
| Total | 17.0 | 16.2 | 15.8 | 15.4 | 15.1 | 16.2 | 16.5 | 16.2 | 15.7 | 15.2 |
| First | 17.1 | 16.4 | 15.9 | 15.5 | 15.2 | 16.3 | 16.6 | 16.3 | 15.7 | 15.1 |
| Recurrent | 16.3 | 14.5 | 14.1 | 14.4 | 13.9 | 14.3 | 14.4 | 15.0 | 15.6 | 15.8 |
