# Supplementary material for: Incidence and case fatality of acute myocardial infarction in Korea, 2011-2020
Source: Epidemiol Health. 2023 Dec 26;46:e2024002. doi: 10.4178/epih.e2024002 (PMC10928467; doi:10.4178/epih.e2024002)
Supplement: Supplementary Material 10. — Age-stratified one-year case fatality of AMI, 2011-2020 (%) [file epih-46-e2024002-Supplementary-10.docx]

Supplementary Material 10. Age-stratified one-year case fatality of AMI, 2011-2020 (%)

| **Age, years** | **Year** | | | | | | | | | |
| --- | --- | --- | --- | --- | --- | --- | --- | --- | --- | --- |
|  | **2011** | **2012** | **2013** | **2014** | **2015** | **2016** | **2017** | **2018** | **2019** | **2020** |
| > 80 | 42.5 | 41.0 | 40.0 | 39.0 | 37.3 | 38.1 | 39.6 | 38.5 | 37.4 | 35.4 |
| 65-79 | 19.4 | 18.6 | 18.2 | 17.3 | 16.9 | 17.9 | 17.8 | 17.3 | 16.5 | 15.7 |
| 40-64 | 6.5 | 5.8 | 5.7 | 5.4 | 5.6 | 6.4 | 6.1 | 5.9 | 5.8 | 6.1 |
