# Supplementary material for: Incidence and case fatality of acute myocardial infarction in Korea, 2011-2020
Source: Epidemiol Health. 2023 Dec 26;46:e2024002. doi: 10.4178/epih.e2024002 (PMC10928467; doi:10.4178/epih.e2024002)
Supplement: Supplementary Material 11. — Positive predictive value of the working definition for AMI event [file epih-46-e2024002-Supplementary-11.docx]

**Supplementary Material 11. Positive predictive value of the working definition for AMI event**

| **Classification of Hospitals** |  | **Characteristics of stroke** | | | | | | |
| --- | --- | --- | --- | --- | --- | --- | --- | --- |
|  | **First** | | | |  | **Recurrent** | | |
|  | **Epidemiological determination** | | **Identification algorithm** | **PPV** |  | **Epidemiological determination** | **Identification algorithm** | **PPV** |
| **Hospital types** |  | |  |  |  |  |  |  |
| Tertiary hospitals | 584 | | 617 | 94.7% |  | 88 | 108 | 81.5% |
| Secondary hospitals | 339 | | 368 | 92.1% |  | 138 | 184 | 75.0% |
| Primary hospitals | 51 | | 121 | 42.1% |  | 0 | 1 | 0% |
| **Total** |  | |  |  |  |  |  |  |
| General | 974 | | 1,106 | 88.1% |  | 226 | 293 | 77.1% |
| Weight 1** | - | | - | 92.0% |  | - | - | 77.8% |
| Weight 2^†^ | - | | - | 92.2% |  | - | - | 78.2% |

**Weighted pooled based on the initial medical institution

†Weighted pooled based on the highest-level medical institution

*PPV, Positive Predictive Value
